# Disseminated fusarium infection after allogeneic hematopoietic stem cell transplantation after CART: A case report

**DOI:** 10.1097/MD.0000000000031594

**Published:** 2022-11-11

**Authors:** Hairong Fei, Xiaodan Liu, Lingjie Sun, Xue Shi, Wei Wang, Hongguo Zhao, Chunting Zhao

**Affiliations:** a Department of Hematology, the Affiliated Hospital of Qingdao University, Qingdao, Shandong, China.

**Keywords:** B-cell acute lymphoblastic leukemia, chimeric antigen receptor-modified T cells, Allogeneic hematopoietic stem cell transplantation, Immunosuppression, Fusarium infection

## Abstract

Fusarium is a conditional pathogen that can cause invasive infection in patients with hematological diseases under immune function.

**Methods:**

A case of recurrent and refractory Philadelphia chromosome-positive acute lymphoblastic leukemia was treated with allogeneic hematopoietic stem cell transplantation after chimeric antigen receptor-modified T cells treatment.

**Results:**

During transplantation, disseminated Fusarium infection occurred, involving the skin, liver, spleen and central nervous system, and the patient eventually died.

**Conclusions:**

Early identification of Fusarium infection based on the characteristic rash and timely antifungal treatment can improve the cure rate.

## 1. Introduction

Chimeric antigen receptor-modified T cells (CART) is an important technology in the field of immunotherapy, which offers hope for patients with recurrent and refractory hematological malignancies, especially acute lymphoblastic leukemia.^[1,2]^ However, serious infections caused by immune deficiency after CART treatment need careful attention of clinicians. Fusarium is a conditional pathogen that can cause invasive infection in patients with hematological diseases and immune deficiency. A case of recurrent and refractory Philadelphia chromosome positive acute lymphoblastic leukemia was treated at our department. After CART treatment, allogeneic hematopoietic stem cell transplantation (HSCT) was bridged. During transplantation, disseminated Fusarium infection occurred, involving the skin, liver, spleen, and central nervous system. The case report is described below.

## 2. Case presentation

The patient was a 14-year-old male with previous history of patent foramen ovale. On May 5, 2018, he was admitted to The Affiliated Hospital of Qingdao University for the first time due to fever, and was diagnosed as acute B-lymphocyte leukemia (com-B-ALL, PH positive) based on the relevant examination. The blood routine test showed white blood cells were 61 × 10^9^/L, and immunotyping showed CD34, CD10, CD19, cyCD79a, TdT, and CD22 expression. The chromosomes were 46, XY. *BCR-ABL* fusion gene was positive, *ASXL1* gene showed p. S1146L mutation. Induction chemotherapy with VTCP (vindesine 1.4 mg/m^2^ for days 1.8.15.22, pirarubicin 20 mg/m^2^ for days 1–3, 15–17, cyclophosphamide 450 mg/m^2^ for days 1, 15, and dexamethasone initial dose of 5 mg/m^2^, which was gradually reduced for 28 d) regimen combined with imatinib was given for 35 days. Bone marrow puncture prompted complete remission, the proportion of abnormal lymphocytes with minimal residual leukemia was < 0.01%, and the BCR/ABL P190 was 0. The patient was administered 4 cycles of consolidation chemotherapy, 4 lumbar punctures and intrathecal chemotherapy. No abnormal white blood cells were found in cerebrospinal fluid. On 4 November 2018, 0.04% abnormal B lymphocytes were found in bone marrow immunophenotyping, and the quantitative detection of BCR/ABLp190 was 1.54%. Replacement of chemotherapy regimens failed to achieve complete remission again, ABL kinase was positive, and T315 I mutation. Bone biopsy on April 24, 2019, showed 72.5% abnormal cells in morphology, 37.39% abnormal cells in flow cytometry, 46, XY, t (9,22) t(9;22)(q34;q11)[10]/46, idem, t(1;10)(p35;q26), t(7;19)(p13;p11)[2]/46, idem, add(1)(q34), add(8)(p11)[1]/46, idem, -3, add(3)(p11), t(5;10)(p11;q11), +mar[1]/46,XY [16]. Mutation detection for gene resistance showed that c. 944C > p. T315I mutation occurred in the ABL1 kinase region of *BCR-ABL1* fusion gene. *BCR-ABL* (p190) gene was 98.64%. The mouse-derived CD19-CART cells were infused on May 9, 2019, after the tumor load was reduced by FTA (cytarabine 0.1 g d1-4 + aclacinomycin hydrochloride 10 mg d1-2 + fludarabine 50 mg d2-4) scheme. Bone marrow smears showed complete remission and MRD negative on day 18 of reinfusion.

**Figure 1.**
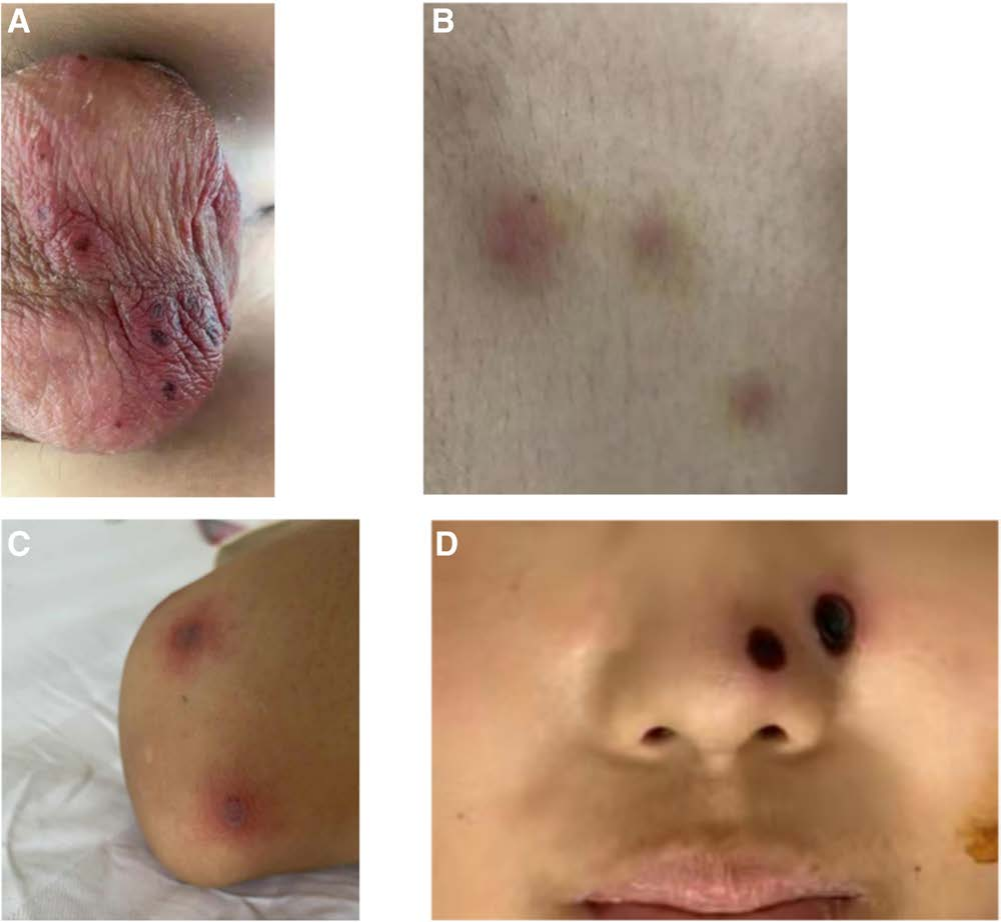
(A) At the beginning of the disease, typical blister-like rash was seen in the scrotum; which gradually developed scabs, forming crater-like changes. (B) Nine days after onset, the skin showed scattered red nodules. (C) During the progressive stage of lesions, pus formed in the center of the rashes and the lesions gradually crusted and became necrotic. (D) During the skin lesion healing period, the rashes gradually became black and necrotic.

**Figure 2.**
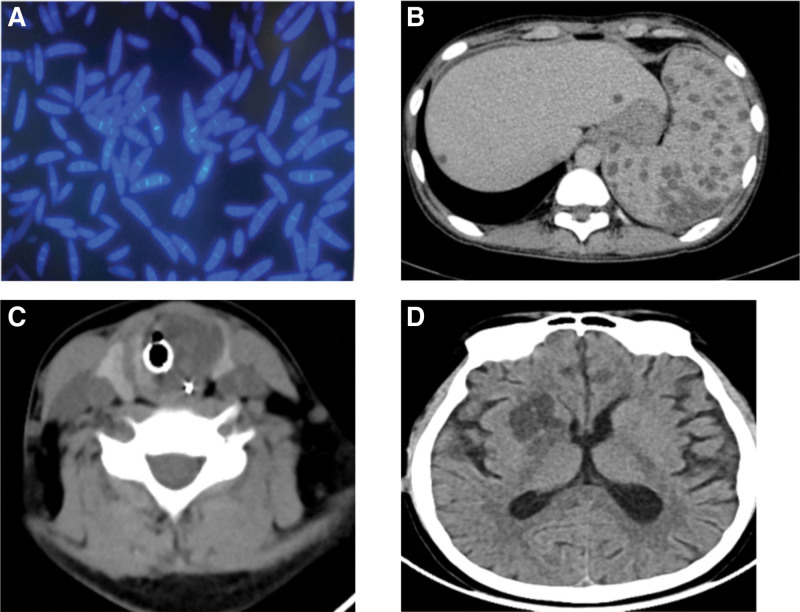
(A) Pus culture showed Fusarium mirror image. (B) Diffuse low-density nodular shadow of the liver and spleen. (C) Neck soft tissue involvement. (D) Intracranial lesions.

The patient found HLA identical donors in the Chinese Bone Marrow Bank. The pretreatment scheme was improved BUCY scheme. Methotrexate (ATG) combined with cyclosporine and short-term ATG was used to prevent graft versus host disease (GVHD), and voriconazole was given to prevent fungal infection. On July 1, 2019, the patient was infused with peripheral blood hematopoietic stem cells (MNC 12.33 × 10^8^/kg, CD34 + 3 × 10^6^/kg) from unrelated donors. Testicular erythema appeared + 1 day after reinfusion, and the right side was indigenous. The erythema was the size of a soybean and was scattered, accompanied by pain and itching, and antiviral therapy was given. The erythema gradually developed blisters, ruptured, followed by scabs (Fig. 1A). Multiple nodules developed in bilateral shoulder, trunk, thigh medial and lateral, and bilateral knee joints in + 9 days, of about 1 cm in diameter, tough, local skin redness, increase in skin temperature, with tenderness (Fig. 1B). The center of the nodules gradually broke, forming crater-like changes (Figs. 1C and 1D); vancomycin 0.5 g q12 h treatment was administered, + 14 days testicular exfoliated skin biopsy showed spores and hyphae, indicative of fungal infection (Candida?). Posaconazole 10 mL bid orally, amphotericin B liposome intravenous drip, and external application of fluconazole treatment was administered. At + 22 days, blood culture and pus cell culture showed Fusarium infection (Fig. 2A), which confirmed disseminated Fusarium infection. Posaconazole combined with amphotericin B liposome antifungal therapy was continued. The patient’s skin lesions improved but he had recurrent fever. At + 44 days, CT showed multiple low density lesions in the liver and spleen (Figure 2B). No liver and spleen biopsy was performed to clarify the condition because the patient had poor megakaryocyte implantation and low platelet count. Since patients with leukemia in complete remission (+ 25 d hematopoietic reconstitution, + 32 d chimerism rate 99.76%, + 38 d bone marrow puncture showed complete remission, *BCR/ABL* fusion gene P190 negative), it was speculated that the intrahepatic and splenic space occupation of the patient was caused by Fusarium infection. The antibiotics were adjusted to amphotericin B combined with voriconazole and meropenem on + 54 days. The skin lesions healed in + 70 days, pain disappeared, leaving scars. The body temperature was normal, and the G experiment gradually decreased. The patient suddenly developed wheezing on + 79 days and went into a coma. The bilateral pupil size was unequal and the light reflex was weakened. Blood gas analysis showed type II respiratory failure, and PCO2 was > 150 mm Hg. After tracheal intubation, his mind was cleared. Craniocerebral CT showed low-density lesions in the right basal ganglia, and anterior feet of the right lateral ventricle were compressed (Fig. 2C). Neck CT showed thickening of the air duct wall and soft tissue density (Fig. 2D). Anti-infection was administered with amphotericin B + voriconazole + tigecycline + meropenem, but the patient’s condition did not improve. The patient’s condition deteriorated on + 94 days and he died on + 95 days after HSCT.

## 3. Discussion

Disseminated Fusarium infection has high mortality and poor prognosis. The mortality rate reported in the literature was 50 and 70%.^[3]^ The patient with disseminated Fusarium infection reported herein had a typical rash and was diagnosed based on bacilli culture, but the final treatment failed due to severe immune deficiency.

Fusarium is a common saprophytic fungus in soil, and it is a conditional pathogen that can cause invasive or localized infection.^[4]^ The severity and duration of immunosuppression in patients with hematological malignancies are the most important factors determining the risk of Fusarium disease.^[5–7]^ Fusarium infection is characterized by invasion of vascular walls, and usually spreads throughout the body by blood circulation, so it can infect all organs of the body. The common sites of disseminated Fusarium infection include lung, blood, skin, and honeycomb.^[8]^ Lesions infected by Fusarium are often manifested as multiple nodular erythemas with tenderness, darkness and pustular necrosis in the center.^[9]^ Skin lesions are specific, and early identification helps with timely targeted antifungal therapy to prevent the spread of infection.

There are no optimal treatment strategies for disseminated Fusarium infection. The results of drug sensitivity test in vitro showed that fluconazole, itraconazole, and fluorouracil had no anti-fungal activity against Fusarium. The commonly used drugs for treatment were high-dose liposomal amphotericin B (>5 mg/kg/d).^[10,11]^ It was reported that 46% of patients improved, but only 13 and 21% of patients survived after 90 days.^[12–14]^ Patients with immunodeficiency show less efficacy. At present, the main treatment is the combination of voriconazole and amphotericin B, especially in cases that show no effect with antifungal drugs alone. Recent European guidelines recommend the use of voriconazole and surgical debridement for the treatment of Fusarium disease and posaconazole as a rescue treatment.^[15]^

CART cells refer to T cells of chimeric antigen receptor (CAR). Through gene transduction, T lymphocytes express specific CAR to construct specific CART cells, so as to specifically recognize target antigens and kill target cells.^[16]^ CART cells have high affinity towards specific tumor antigens and high efficiency in killing tumor cells expressing antigens in vitro and in vivo. The adverse reactions of CD19-CART treatment include tumor dissolution syndrome, cytokine storm, macrophage syndrome, neurotoxicity, and B cell loss.^[17–19]^ CD19 antigen is expressed on the surface of all B lymphocytes except plasma cells. After treatment with CD19-CART cells, CD19 positive cells in the patients were targeted and removed, resulting in humoral immune dysfunction, increased risk of infection, including some rare pathogen infections.

In addition to the immune deficiency after CAR-T treatment, this patient also had disseminated Fusarium infection, which was related to unrelated donor allogeneic HSCT and GVHD prevention treatment. The patient was pretreated with modified BUCY regimen, ATG combined with cyclosporine and short-term ATG to prevent GVHD. After pretreatment and bone marrow suppression, he developed granulocyte deficiency. Removal of T lymphocytes after ATG treatment worsened the degree of immune deficiency. Therefore, disseminated Fusarium infection occurred after voriconazole preventive antifungal therapy. The patient had atypical skin lesions in the early stage, was not administered other anti-fungal drugs in time, and the infection occurred in the early stage of transplantation when his hematopoietic and immune functions were not restored, which provided an opportunity for the spread of Fusarium, eventually leading to liver, spleen, central involvement, and death. Due to thrombocytopenia and other reasons, we did not perform the liver–spleen and intracranial pathology. However, we can infer from the course of disease that the liver–spleen and intracranial occupations of the patient were caused by disseminated Fusarium infection.

## 4. Conclusion

In patients with bridge transplantation similar to CART treatment, especially those who need to use ATG to prevent GVHD, the risk of opportunistic infections should be fully recognized in order to facilitate early identification and timely intervention. Early identification of Fusarium infection according to characteristic rash and timely antifungal treatment can improve the cure rate.

The authors obtained informed consent from the family members of the patient for publication of this case report and associated images.

## Author contributions

**Conceptualization:** Hairong Fei, Xiaodan Liu.

**Data curation:** Hairong Fei, Xiaodan Liu, Lingjie Sun, Xue Shi, Wei Wang, Hongguo Zhao, Chunting Zhao.

**Formal analysis:** Chunting Zhao, Hairong Fei, Xiaodan Liu, Lingjie Sun, Hongguo Zhao.

**Investigation:** Xue Shi.

**Methodology:** Chunting Zhao, Wei Wang.

**Resources:** Lingjie Sun.

**Writing—original draft:** Hairong Fei, Xiaodan Liu.
